# Epidemiological Investigation of Canine Mammary Tumors in Mainland China Between 2017 and 2021

**DOI:** 10.3389/fvets.2022.843390

**Published:** 2022-06-22

**Authors:** Hui-Hua Zheng, Chong-Tao Du, Chao Yu, Yu-Zhu Zhang, Rong-Lei Huang, Xin-Yue Tang, Guang-Hong Xie

**Affiliations:** Department of Clinical Veterinary Medicine, College of Veterinary Medicine, Jilin University, Changchun, China

**Keywords:** canine mammary tumors, epidemiology, clinical, benign, malignant

## Abstract

Epidemiological studies enable us to analyze disease behavior, define risk factors, and establish fundamental prognostic criteria. This study aimed to determine the epidemiological and clinical characteristics of canine tumors diagnosed during the years 2017–2021. The results showed that canine mammary tumors were the most common tumors, and their relative incidence for 5-years-total was 46.71% (504/1,079), with 48.41% (244/504) of benign, and 51.59% (260/504) of malignant. Pure breeds accounted for 84.13% (424/504) of submissions, and adult female dogs (9–12 years old) were most frequently involved, followed by 5–8-year-old females. Remarkably, 2.58% (13/504) occurred in the male dogs. In addition, a high prevalence of mammary tumors (77.38%, 390/504) was diagnosed in unneutered dogs, and different incidence rates were observed in different regions (Northeast, Southeast, Northwest and Southwest China). For clinical factors, the tumor size ranged from 0.5 to 28 cm, with the 0–5 cm being the most common tumor size (47.82%, 241/504), and malignant tumors (4.33 ± 2.88 cm, mean ± SD) were bigger than benign ones (3.06 ± 1.67 cm, mean ± SD) (*p* < 0.001). The incidence of single tumor (55.36%, 279/504) was higher than that of multiple tumors in dogs, while the latter had a higher incidence of malignant tumors (74.67%, 168/225). According to this study, we also found that canine mammary tumors were more common in the last two pairs of mammary glands. In addition, multiple linear regression analysis showed that there was linear significant relationship between three independent variables (age, tumor size, and tumor number) and histological properties of canine mammary tumor [(p>|*t*|) < 0.05]. This is the first retrospective statistical analysis of such a large dataset in China to reveal the link between epidemiological clinical risks and histological diagnosis. It aids in the improvement of the host's knowledge of canine tumor disorders and the early prevention of canine mammary tumors.

## Introduction

Breast cancer (HBC) is one of the most commonly diagnosed cancers in women worldwide, and it is the second leading cause of patient mortality (1). In recent years, an increasing number of household pets, particularly dogs, have been raised as spiritual sustenance for humans. As one of the companion animals, domestic dogs' environments and lifestyles are similar to those of humans, making them valuable models. Canine mammary tumors (CMTs) are the most common tumors in female dogs, with around 50% of cases being malignant, and the morbidity being three times that of women (2–5). CMTs are usually observed in 5-year-old and older dogs, and their incidences are also connected with the breeds of dogs, such as Maltese, Yorkshire terriers, Shih Tzu, Dachshunds, cocker spaniels, toy poodles, German shepherds, and mixed-breed dogs (6–8). Canine mammary carcinomas exhibit a high degree of variety in terms of shape and biology, which has been a hot topic of in-depth investigation for several decades. Histologically, mammary tumors are generally classified as benign or malignant tumors (9). Mammary gland tumors share common features between dogs and humans, so dogs are ideal models for human breast cancer research and comparative studies on breast cancer prognosis and treatment (10, 11). Although some molecular and biological characteristics of CMTs are similar to those of HBC, there are differences between them, such as the fact that canine mammary sarcomas usually undergo multi-differentiation (bone, cartilage, and fat), which is not a common phenomenon in humans, and sarcomas that resemble HBC (malignant) phyllodes tumors are quite rare in dogs (12). As a result, despite extensive research on HBCs, there is a need for additional research on CMTs.

According to the 8th International Pet Industry Summit Forum, China has an estimated 50.85 million dogs, making it the country with the most dogs. Given the size of the dog population and the lack of nutritional information, the prevalence of canine mammary tumors may have increased in recent years. The canine mammary tumor is a multifactorial disease, meaning that multiple factors contribute to its occurrence and progression (13). For instance, epidemiological, clinical, and histological factors all play a role in tumor behavior. Breed, age, gender, and spayed status were also reported in the literature as contributing factors (2, 13–15). Although canine mammary tumors can occur in any breed of dog, pure breeds appear to be more prone to tumor development (2, 13–16). Canine mammary tumors are more common in middle- and older-age dogs with a significant morbidity between the ages of 8 and 11 years (13). Furthermore, a study reported that performing ovariohysterectomy on dogs before their first estrus may reduce the possibility of tumor formation, and a more recent retrospective study found that the performing ovariohysterectomy with resection of benign tumors can obviously decrease the incidence of new tumors (17, 18). Clinically, the tumor size strongly impacts on prognosis of canine mammary tumors, which dogs with larger tumors have a poorer prognosis than that with smaller tumors in both remission and survival (19).

Food is typically regarded as one of the factors which can predispose to tumor development (20, 21), and the diet management and potential food pollutant levels vary in different geographical regions in China, such as the Northeast China, Southeast China, Northwest China, and Southwest China. Hence, the epidemiological risk of tumor is probably associated with the geographical regions. As for clinical factors, the number of tumors is generally related to their histological characteristics (benign or malignant). Some studies indicate that the status of the regional lymph node plays an important role in the survival of dogs with mammary tumors (4, 22) and five pairs of canine mammary glands can drain to different lymph nodes, which can further conduct tumor metastasis by lymph circulation. It implies that tumor risk varies with different locations of canine mammary glands. The relationship between these factors and the occurrence of canine mammary tumors has not been well established. To our best knowledge, the impact of epidemiological, clinical, and histological factors on canine mammary tumors has not been reported in China. Thus, in this study, we measured the relative frequency of CMTs in different geographical regions of mainland China, and also analyzed the prevalence of common canine tumors in clinical settings, such as mammary gland, skin, perianal, vaginal, oral cavity, testis, and other tumors. In addition, we examined the association between epidemiological–clinical risks (the breed, age, spayed status, gender, and geographical region—the tumor size, number, and location) and histological diagnosis in a comprehensive retrospective statistical analysis of canine mammary tumors. This study will contribute to our understanding of canine cancers, particularly in terms of early detection and prevention of canine mammary tumors.

## Materials and Methods

### The Collection of Data

This study focused on reviewing canine tumor data collected from several animal hospitals, therefore, the permission from the University's Animal Care Ethics Committee was not required. The data included breed, age, gender, spayed status, body shape, and geographical region, the histopathological diagnosis, size, number, and location of tumor. During the period September 2017–December 2021, 504 cases of spontaneous mammary tumors in dogs aged 1–19 years of various breeds were collected from mainland China. The procedure for pathological diagnosis of CMTs was as follows: tissues submitted in 10% neutral buffered formalin were embedded in paraffin wax, and sections (5 μm thick) were cut and routinely stained with H and E for histological examination. The histopathological classification was based on the Surgical Pathology of Tumors of Domestic Animals, Volume 2: Mammary tumors (9).

### Survey of Canine Tumors

Each dog's occurrence of canine tumors was noted. One thousand and seventy-nine cases were gathered and examined to better understand the incidence rate of various types of canine tumors in mainland China, and the body sections involved in producing tumors included the skin, perianal, vagina, oral cavity, testis, and others of dogs. Some general information on affected dogs (gender and spayed status) was collected to further investigate the epidemiology of canine tumors.

### Epidemiological of Canine Mammary Tumor

To determine whether there was an association between epidemiological factors (age, breed, gender, spayed status, and geographical region) and tumor diagnosis and clinical characteristics (tumor size, number, and location), they were evaluated in conjunction with histological diagnosis. For further analysis, the breed was recorded as pure breed or mixed breed.

**Figure 1:**
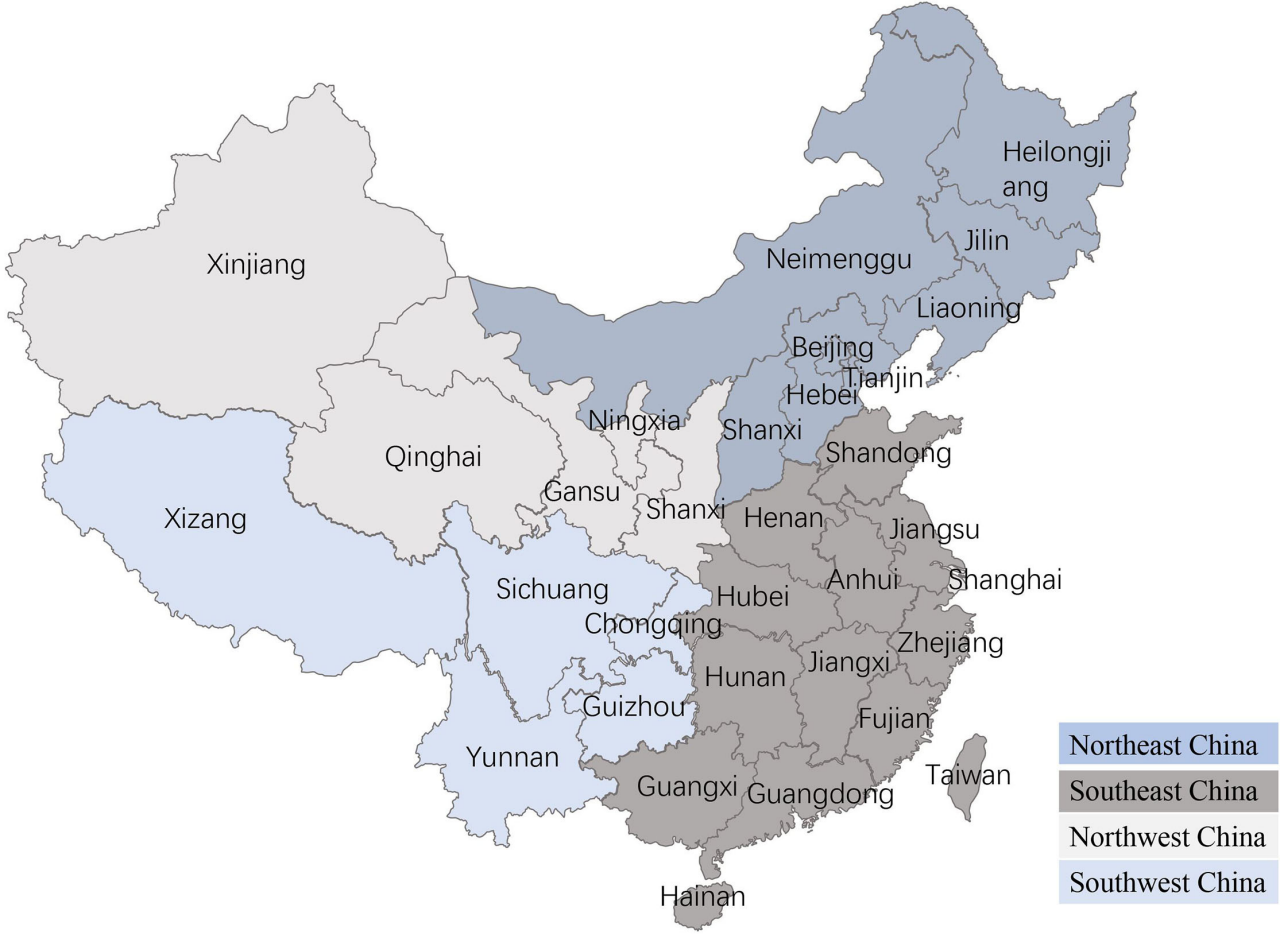
Regional distribution in China. The mainland China is consists of four parts: Northeast China, Southeast China, Northwest China, and Southwest China.

Age was treated as a numerical variable (in years), with four categories (0–4 years, 5–8 years, 9–12 years, and >13 years), and tumor size was classified into three categories based on the World Health Organization (WHO) tumor system, including T1 <3 cm, T2 = 3–5 cm, and T3 > 5 cm (4). Breeds were split into three classes: small (<35 cm), medium (35–50 cm), and large (>35 cm) based on cross height, and the breeds were congruent with the Federation Cynologique Internationale (FCI) Breeds Nomenclature. The dogs included in the study were from various regions: the Northeast China, Southeast China, Northwest China, and Southwest China (Figure 1). Because tumor numbers vary from dog to dog, dogs with mammary tumors were classified into two groups based on the number of tumors per dog: single tumor (*n* = 1) and multiple tumors (*n* > 1). The locations of canine mammary tumors were treated as five classes (the first pair, the second pair, the third pair, the fourth pair, and the fifth pair in the mammary gland). According to Misdorp et al., canine mammary tumors were classified as benign and malignant, and the degree of malignant was divided into grades I, II, and III (23).

### Multiple Linear Regression Analysis and Machine Learning Model

Multiple linear regression analysis was carried out to assess the influence of the various covariates (age, breed, gender, spayed status, geographical region, tumor size, number, and location) on the tumor properties by using ordinary least square (OLS) on python code. In the multiple linear regression model, four formulas were involved, such as *y* = β_0_+β_1_*X*_1_+β_2_*X*_2_+…+β_p_*X*_p_+ε, $\overset{n}{\underset{i=1}{\sum }}{\left(yi-\bar{y}\right)}^{2}=\overset{n}{\underset{i=1}{\sum }}{\left(ŷ-\bar{y}\right)}^{2}+\overset{n}{\underset{i=1}{\sum }}{\left(y_{i}-\;ŷ\right)}^{2}$, SST = SSR+SSE, *F* $=\frac{\frac{SSR}{p}}{\frac{SSE}{\left(n-p-1\right)}}$ and *R*^2^ = $\frac{SSR}{SST}$ = 1 – $\frac{SSE}{SST}$. The procedures were as follows: we assumed that the linear regression equation between random variable y and general variables consisted of ×1, × 2… × *p* was *y* = β_0_ + β_1_*X*_1_+β_2_*X*_2_+…+β_p_*X*_p_+ε, and among them, β_1_,β_2_,…,β_p_ were the regression coefficient and β_0_ was a regression constant. According to the data in this study, the form of matrix *y* = *X*β+ε was obtained, and then the significance of the regression equation needed to be tested, including the *F*-test, *t*-test and goodness-of-fit test. *F*-test and *t*-test were used to determine whether these general variables from the whole and itself had a significant impact on the random variable y, respectively, and the value of goodness of fit, *R*^2^ was between 0 and 1, which was directly proportional to the regression fitting effect.

### Statistical Analyses

The analysis of data was performed using descriptive statistics, as well as bivariate and multivariate analyses. Data were analyzed using Prism 7.0 (Graph Pad Inc., USA), and the value of *p* < 0.05 was considered significant.

## Results

### Prevalence of Different Types of Canine Cancers

In this study, a total of 1,079 canine tumors including 504 canine mammary tumors were included. The prevalence of tumorigenesis in different tissues was 46.71% (504/1,079), 34.11% (368/1,079), 3.71% (40/1,079), 2.78% (30/1,079), 5.38% (58/1,079), 3.52% (38/1,079), and 3.79% (41/1,079) in the mammary glands, skin, perianal, vagina, oral cavity, testis, and others, respectively. To further analyze the relationship between characteristics of dogs and tumors, gender and spayed status of ill dogs were collected, and the results are displayed in Table 1. Canine mammary tumors were most common in unneutered female dogs, accounting for approximately 75.20% (379/504) of dogs with CMT. The probability of cutaneous and oral tumors in female dogs (48.37%, 178/368) did not differ significantly from that in male dogs (51.64%, 190/368), and there was no significant difference between intact dogs and castrated/neutered dogs (*p* > 0.05). Male dogs were more likely to develop perianal tumors (85%, 34/40), although the probability of tumor development in castrated dogs (52.94%, 18/34) was no different from that in non-castrated dogs (47.06%, 16/34). Among reproductive tumors, the vaginal tumor happened only in female dogs (100%, 30/30) with 46.67% (14/30) of neutered dogs and 53.33% (16/30) of non-neutered dogs, and the testicular tumor occurred only in male dogs without castration (100%, 38/38).

**Table 1:** Distribution of tumor growth sites in dogs.

**Location of tumor**	**Number of cases**	**Male**		**Female**		**Percentage**
		**Castration**	**No castration**	**Sterilization**	**No sterilization**	
Mammary gland	504	11	2	112	379	46.71%
Skin	368	104	86	99	79	34.11%
Perianal	40	18	16	0	6	3.71%
Vagina	30	0	0	14	16	2.78%
oral cavity	58	14	18	14	12	5.38%
Testis	38	0	38	0	0	3.52%
Other	41	8	12	12	9	3.79%
Total	1,079	155	172	251	501	100%

### Prevalence of Canine Mammary Tumors During the Research Period

**Figure 2:**
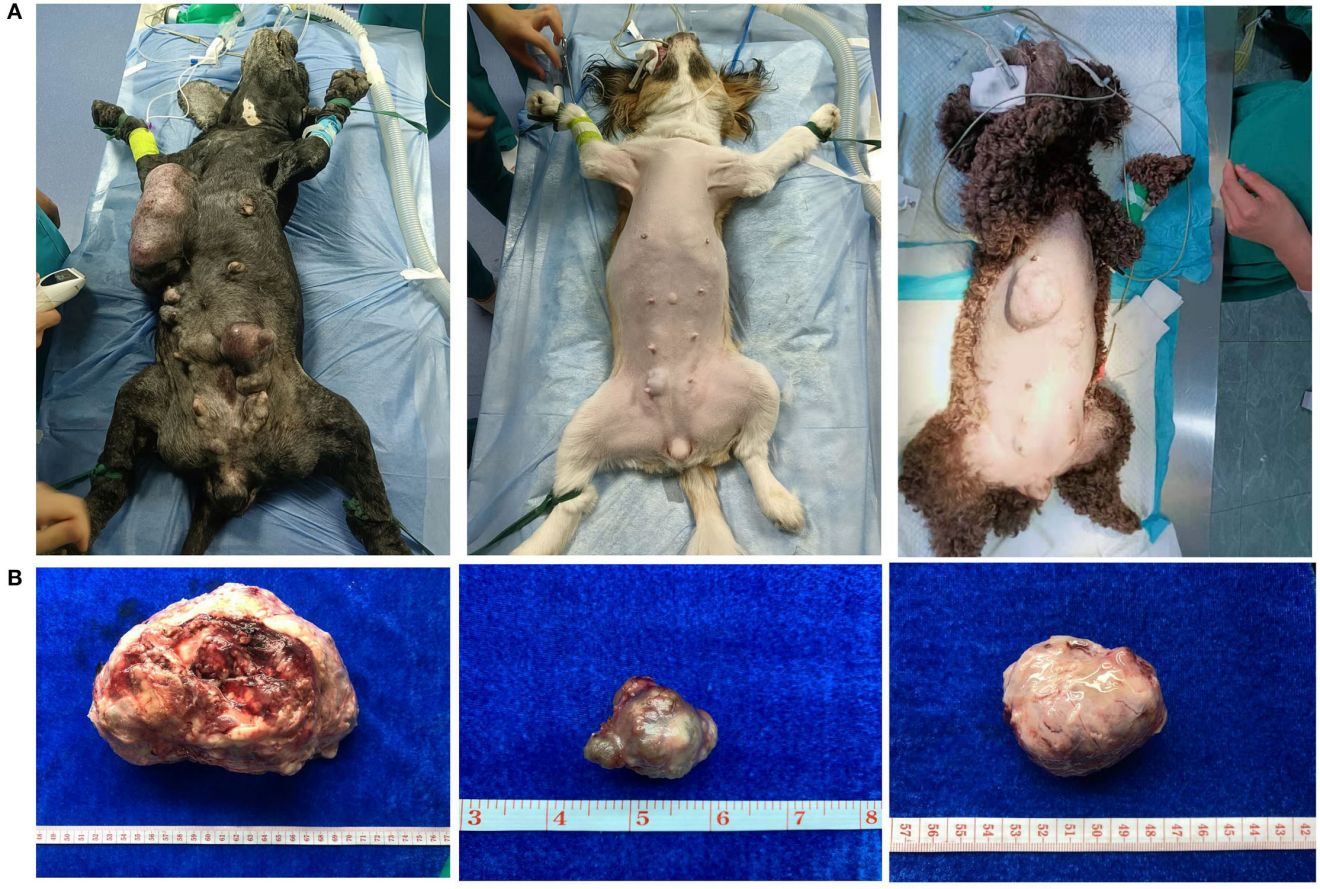
Clinical appearance of canine mammary tumors. **(A)** Preoperative appearance of dog: diseased dogs were mainly characterized by localized swelling, hard texture, painless, and itchless of mammary gland and surrounding subcutaneous tissue. **(B)** Postoperative picture of tumor: the tumor size is variable.

**Figure 3:**
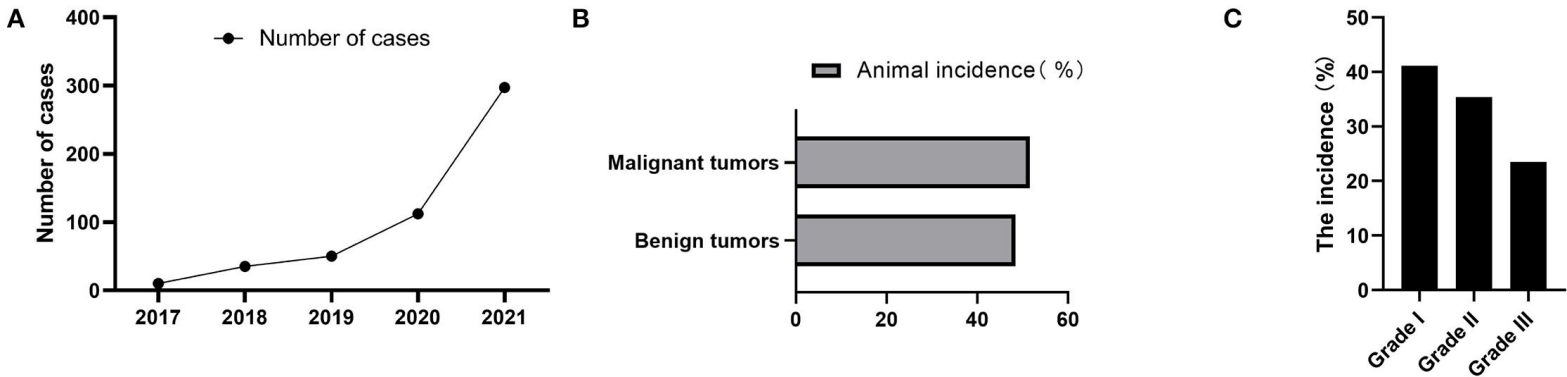
Annual cases and pathological characteristics of canine mammary tumors. **(A)** The number of dogs suffered from CMT during 2017 to 2021. **(B)** Relative incidence of canine mammary tumors according to biological behavior at five-years-total. **(C)** The 5-year-total prevalence of three histological malignant categories.

CMT was the most common type of canine tumor in this study, and it was characterized by localized swelling, hard texture, painless, and itchless of the mammary gland and surrounding subcutaneous tissue, with varying sizes (Figures 2A,B). The number of cases increased year by year from 2017 to 2021, and yearly incidence rates were 22.72% (10/44), 44.74% (34/76), 61.45% (51/83), 54.89% (101/184), and 44.51% (308/692), respectively (Figure 3A). Based on the histopathological classification of these lesions throughout the study, 48.41% (244/504) were benign tumors, and 51.59% (260/504) were malignant tumors (Figure 3B). According to the three histological malignant categories proposed by Pena et al., the prevalence for the 5-year-total of malignant tumors classified as grades I, II, and III was 41.15% (107/260), 35.39% (92/260), and 23.46% (61/260), respectively (Figure 3C).

### The Relationship Between Epidemiological Factors and Canine Mammary Tumors

#### The Association of Breed With Canine Mammary Tumors

**Figure 4:**
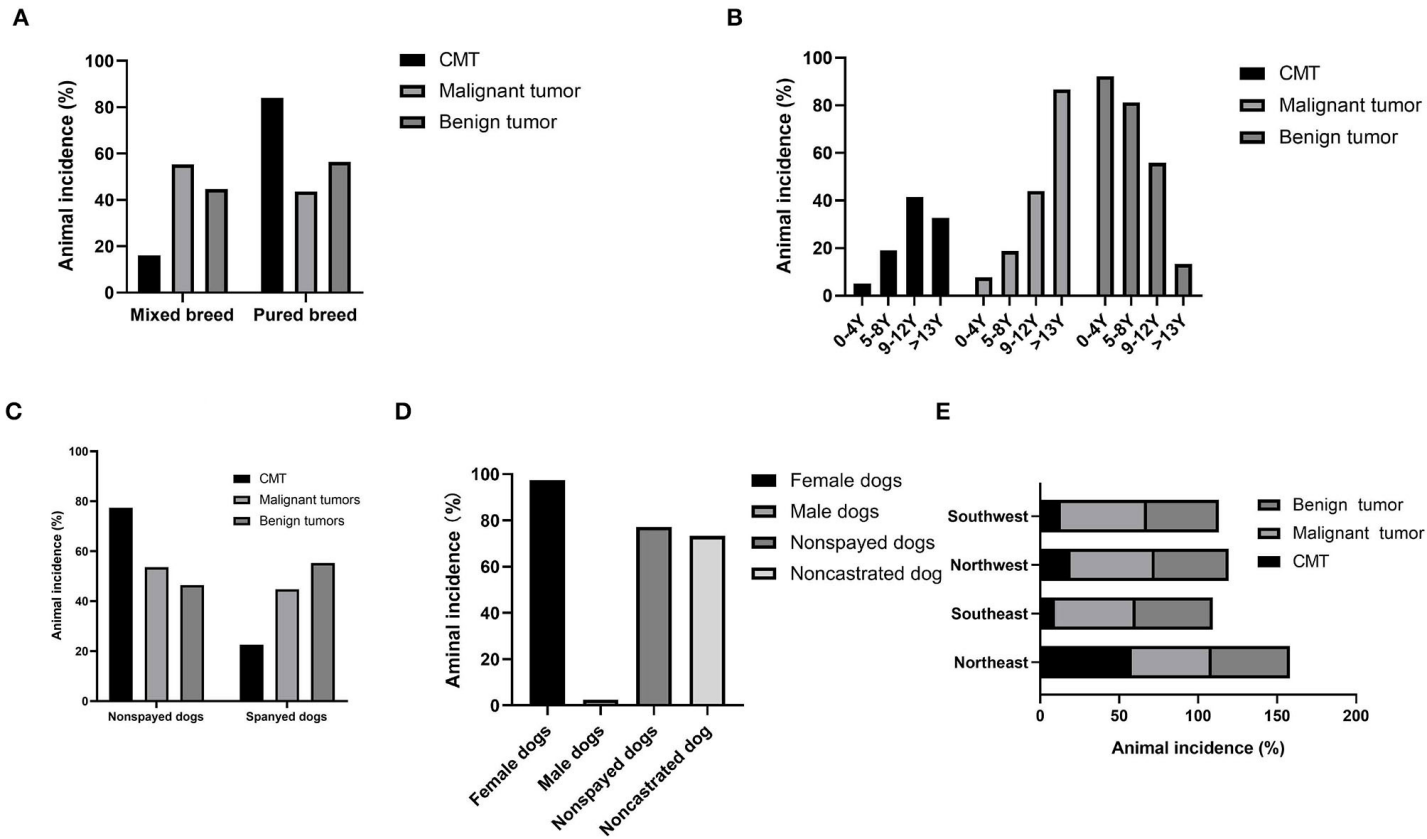
Statistical analysis of pathogenic factors of canine mammary tumors. Dogs diagnosed with benign or malignant neoplasm. **(A)** Percentage of dogs showing canine mammary tumors and classified according to their breed group. **(B)** Age group distribution. **(C)** Distribution of sterilization status of diseased dogs. **(D)** Gender group distribution. **(E)** Distribution of regions of diseased dogs.

A total of 84.13% (424/504) of CMTs were observed in pure breed dogs, mostly represented by small dogs, while the remaining 15.87% (80/504) were observed in mixed breed dogs (Figure 4A). Benign tumors occurred predominantly in small dogs, with poodles the most represented (36.07%, 88/244), and poodle dogs accounted for 28.46% (74/260) of malignant tumors, while there was no statistically significant association between body shapes of dogs and the prevalence of benign and malignant tumors, which was consistent with the study by Pena et al. (24).

#### The Association of Age With Canine Mammary Tumors

Dogs with mammary tumors had an average age of 10.53 ± 3.41 years (mean ± SD). Among them, dogs with malignant mammary tumors were older (12.50 ± 2.63 years, mean ± SD) than those with benign tumors (8.43 ± 2.88 years, mean ± SD) (*p* < 0.001). There were four age groups: 0–4 years, 5–8 years, 9–12 years, and >13 years (Figure 4B), and 62.10% (313/504) of the canine mammary tumors occurred in the age groups of 5–12 years. In addition, the age group of 9–12 years has the highest incidence rates of both benign tumors (60.99%, 111/182) and malignant tumors (39.01%, 71/182).

In addition, the median age of dogs suffering from benign tumors, whether simple or multiples, was younger when compared to those suffering from simple malignant and malignant special types, whereas simple malignant occurred at an older median age than non-simple malignant. None of the three histological malignant categories established by Pena et al. showed a significant difference in this study (24).

#### The Association of Spayed Status With Canine Mammary Tumors

According to statistics, non-spayed dogs accounted for 75.60% (381/504) of canine mammary tumors, while spayed dogs accounted for the remaining 24.40% (123/504) (Figure 4C). Among non-spayed dogs with canine mammary tumors, 47.51% (181/381) had benign and the remaining 52.49% (200/381) had malignant tumors. The spayed status was not significantly different from the three histological categories in Pena's study (24).

#### The Association of Genders With Canine Mammary Tumors

In the present study, 97.42% (491/504) of dogs with mammary tumors were female dogs, consistent with the observations in the previous study (4, 14). Among the female afflicted, non-spayed dogs were predominant (77.19%, 379/491) (Figure 4D). Remarkably, canine mammary tumors were also diagnosed in male dogs, although much rarer with only 2.58% (13/504) of the total. The gender difference of all types of tumors is much less, 73.31% (791/1,079) for females and 26.69% (288/1,079) for males. The three histological malignant categories proposed by Pena et al. have no significant difference in female and male dogs (24).

#### The Association of Geographical Regions With Canine Mammary Tumors

The prevalence of canine mammary tumors for a total of 5 years was 58.13% (293/504), 9.33% (47/504), 19.44% (98/504), and 13.09% (66/504) in the Northeast China, Southeast China, Northwest China, and Southwest China, respectively, and the incidences of malignant tumors were 56.9% (148/260), 9.2% (24/260), 20.0% (52/260), and 13.9% (36/260) in the corresponding regions (Figure 4E). It was worth noting that the highest incidence of 58.1% (293/504) was observed in Northeast China, and the yearly incidence rates from 2017 to 2021 were 1.02% (3/293), 3.75% (11/293), 6.14% (18/293), 22.87% (67/293), and 49.49% (194/293), respectively. Besides, the diagnostic rates of canine mammary tumors in different regions and years were variable. For instance, the diagnostic rates of canine mammary tumors in Northwest China from 2017 to 2019 averaged approximately 48.40% (16/33), and 50.75% (34/67) in 2020, and 49.22% (95/193) in 2021; in Southwest China, the diagnostic rates between 2020 and 2021 were higher than 20.00%, but the diagnostic rates during 2017–2019 were lower than 10.00%; in Southeast China, the diagnostic rates between 2017 and 2020 ranged from 8.51% (4/47) to 19.15% (9/47), and approximately 40.00% in 2021; in Northeast China, the diagnostic rates in 2017–2019 were as low as 10.12%, but that of 2021 was higher than 30.00%. The incidence rate could be related to the different feeding and management methods, biological safety measures, and geographical location.

### Clinical Characterizations of Canine Mammary Tumors

#### Canine Mammary Tumor Sizes

Tumor size is thought to be the most clinically prognostic-related factor. The tumor size ranged from 0.1 to 3 cm (1.75 ± 0.59 cm, mean ± SD), and malignant tumors (1.99 ± 0.60 cm, mean ± SD) were generally bigger than benign ones (1.65 ± 0.56 cm, mean ± SD), which was a significant difference between malignant and benign tumors (*p* < 0.001). Moreover, the numbers of dogs diagnosed as grades I, II, and III were 102, 98, and 60, respectively (24), and the size of tumors from large to small was grade III (5.28 ± 3.90, mean ± SD), grade II (4.38 ± 2.52, mean ± SD), and grade I (3.78 ± 2.07, mean ± SD), respectively. Interestingly, their sizes were 0.2–30 cm, 0.3–13 cm, and 1–10 cm, with the largest size in the grade I group.

According to the WHO tumor size system, 37.70% (190/504) of canine mammary tumors were T1 (<3 cm), 44.05% (222/504) were T2 (3–5 cm), and 18.25% (92/504) were T3 (more than 5 cm) (4). Surprisingly, a high prevalence of tumors with a size of 0–5 cm (81.75%, 412/504) was detected in ill dogs, indicating that canine mammary tumors were frequently T1 and T2. Remarkably, 28.42% (54/190) of the malignant tumors were T1, and 71.58% (136/190) were categorized as simple carcinoma. In addition, the diagnostic rates of T2 and T3 neoplasms based on the malignant tumors were 64.86% (144/222) and 6.74% (62/920), respectively.

#### Tumor Numbers per Dog in Canine Mammary Tumors

The number of tumors in a dog ranged from one to more. This study divided it into two groups: single tumor (*n* = 1) and multiple tumors (*n* > 1). There were 55.36% (279/504) of single tumor and 44.64% (225/504) of multiple tumors, with the former having a lower incidence rate of malignant tumors (48.76%, 136/279) than the latter, which had a higher incidence rate of malignant tumors (74.67%, 168/225) (*p* < 0.05). Considering the relationship between tumor number and sizes of dogs, further statistical analysis revealed that 55.11% (151/274), 51.56% (66/128), and 56.86% (58/102) of single tumor were observed in the small, medium, and large dogs, respectively, and the prevalence of multiple tumors in three sizes of dogs was 44.89% (123/274), 48.43% (62/128), and 43.14% (44/102). Interestingly, this study found that multiple tumors were more common in dogs over 8 years old, with 51.15% (200/391) of the incidence.

#### The Association of Mammary Gland Locations With Canine Mammary Tumors

According to the data analysis in this study, 5.76% (29/504), 10.31% (52/504), 27.38% (138/504), 30.56% (154/504), and 25.99% (131/504) of CMTs occurred in the first to the fifth pair of diseased dogs' mammary gland, respectively. It was more common in the last two pairs of mammary glands, which was consistent with earlier research (15). In the last two pairs of mammary glands, the morbidities of benign and malignant tumors were 45.65% (126/276) and 54.35% (150/276), respectively. The three histological categories had no significant difference in the malignant tumor in the last two pairs of mammary glands (*p* > 0.05).

### Multiple Linear Regression Analysis and Machine Learning Model

**Figure 5:**
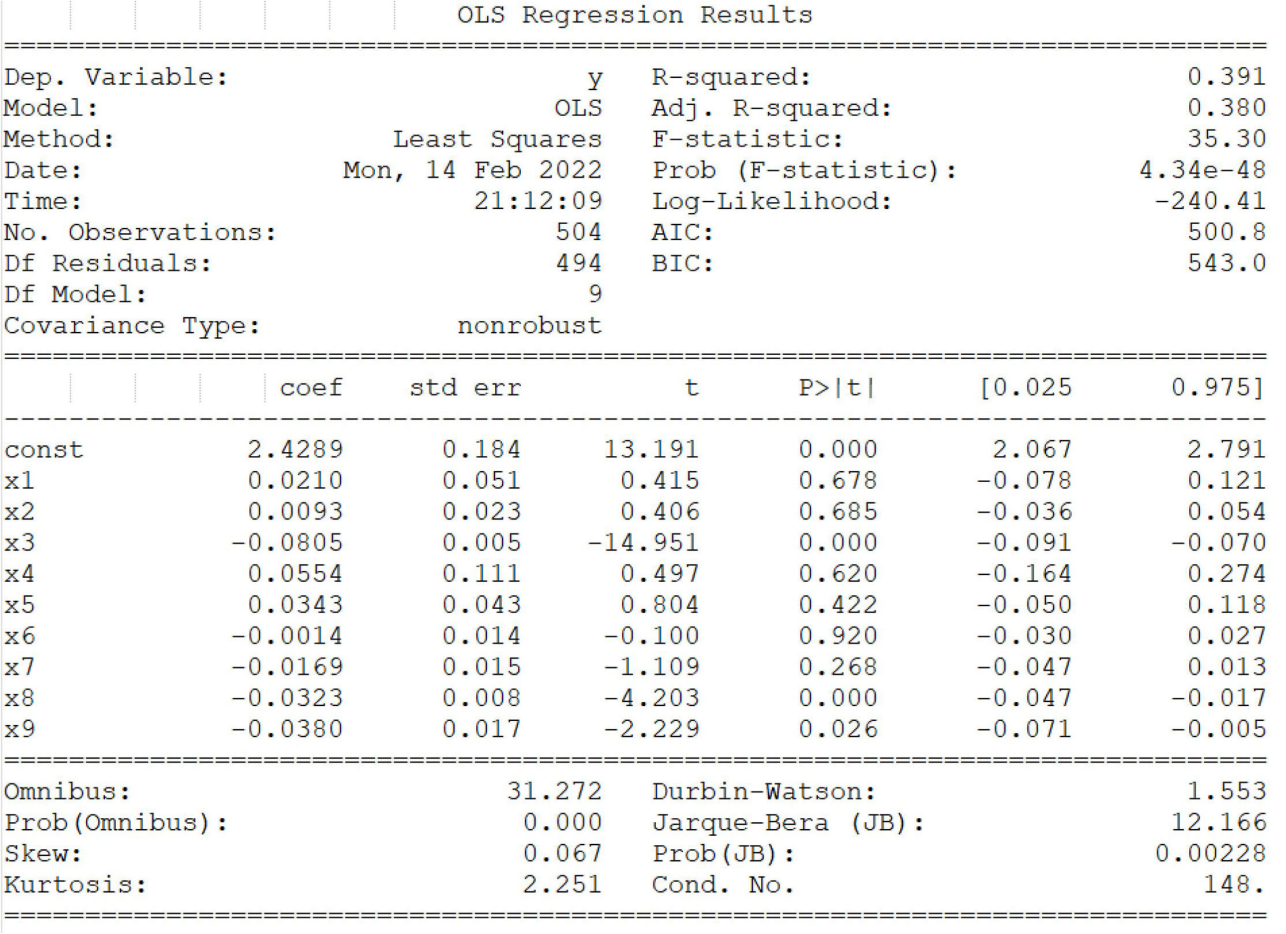
Multiple linear regression analysis and machine learning model. *F* test and *t*-test were assessed whether these general variables from the whole and itself had the significant impact on the random variable *y*, respectively, and the value of goodness of fit, *R*^2^ were between 0 and 1, which was directly proportional to the regression fitting effect. The factors (breed, body shape, age, gender, spayed status, geographical region, tumor location, tumor size, and number) are represented by independent variables ×1, ×2, ×3, ×4, ×5, ×6, ×7, ×8, ×9, respectively. The linear regression equation *y* = 2.4289+0.210* ×1+0.0093* ×2-0.0805* ×3 + 0.0554* ×4+0.0343* ×5-0.0014* ×6-0.0169* ×7-0.0323* ×8-0.0380* ×9. Note: * represents “coef” of corresponding variables.

To analyze the relationship between epidemiological, clinical characteristics, and histological properties of canine mammary tumors. The multiple linear regression analysis was performed on tumor histological properties with OLS by python code, and these factors (breed, body shape, age, gender, spayed status, geographical region, tumor location, tumor size, and number) were used as independent variables. First, these independent variables were pretreated due to the high volume of original data, and then a multiple linear regression model was generated, with the linear regression equation *y* = 2.4289+0.210^*^ × 1+0.0093^*^ × 2-0.0805^*^ × 3 +0.0554^*^×4+0.0343^*^×5-0.0014^*^×6-0.0169^*^×7-0.0323^*^ × 8-0.0380^*^ × 9 (Figure 5). In Figure 5, we mainly analyzed three parameters, namely, “coef”, “*t*” and “*P*>|*t*|”. Among them, “coef” is the regression coefficient, “const” is the regression constant, and “*P*>|*t*|” is mainly used to judge the linear significant relationship between each independent variable and the multiple linear regression model. Results showed that there was a linear significant relationship between three independent variables (age, tumor size, and tumor number) and the histological properties of canine mammary tumor ((*p*>|*t*|) < 0.05). The value of Prob (*F*-statistic), that is, *p*-value was 4.34*e*−48, indicating that the multiple linear regression equation was significant. In other words, there was a significant linear relationship between *y* and ×1, ×2,……, ×8, ×9, but it was noteworthy that the nine variables ×1, ×2,……, ×8, ×9 were regarded as a whole.

## Discussion

As a companion animal closely related to humans, the living environment of dogs is highly similar to that of human beings, and the health of dogs is a very serious concern globally. Due to the multifaceted impacts, there is no complete independent dog health research system in China (25). With the expansion in the number of dogs and the extension of life in recent years, many diseases have increased, with tumor being one of the most frequent, which has posed a significant problem for veterinary oncology professionals. In this study, the prevalence rates of tumorigenesis in various tissues including the mammary gland, skin, perianal area, vagina, oral cavity, testis, and other tissues of dogs were measured, and it was identified that mammary tumors are the most clinically diagnosed, consistent with previous studies (15, 26). Besides, there was no significant difference in the risk of skin and oral tumors between female and male dogs (*p* > 0.05), and their prevalence rates were independent of the intactness of dogs. This could be due to the most common causes of oral tumors being periodontitis, poor oral hygiene, inflammation, and chronic microbial infections, none of these is expected to be gender-related (27). The perianal tumor was prevalent in male dogs, which was associated with tumor growth features. The formation of these tumors appeared to be related to hormones (28). Not surprisingly, gender disparities in reproductive cancers are the most noticeable, such as the vaginal tumor and the testicular tumor.

As the most common tumor, the number of dogs with CMT increased from 2017 to 2021, which could explain the increase in the number of dogs raised as a result of economic development. Its annual incidence rates were 22.72% (10/44), 44.74% (34/76), 61.45% (51/83), 54.89% (101/184), and 44.51% (308/692), correspondingly, and the incidence rate was falling after 2019, possibly due to increased public awareness of pet breeding. The tumor prevalence increased with age, and the top three canine tumor types for 5-year-total were mammary gland, skin, and perianal, with CMT accounting for 46.71% (504/1,079) of tumors.

In human cancer, breast cancer is the most common cancer worldwide (29). Canine mammary tumors are frequently employed as a preliminary study in breast cancer research. CMT, as one of the most prevalent tumors, poses a significant threat to the life and health of dogs, and its treatment and prevention complicate veterinary clinical practice. Despite this, veterinarians have made significant efforts in current research to enhance the early diagnosis and life prospects of dogs with CMTs. Much of the research focused on the elements that contribute to CMT formation (2, 14–16, 26, 30, 31). However, there is yet to be a systematic retrospective statistical analysis in China that links the breed, age, spayed status, gender, tumor size, tumor number, tumor location, and geographical region to the histological diagnosis.

In this study, 51.59% (260/504) of CMTs were benign tumors, and 48.41% (244/504) were malignant tumors during the years of study. Among malignant tumors, grades I, II, and III were 35.25% (86/244), 40.2% (98/244), and 24.6% (60/244), respectively. These results confirmed what has been reported in the literature and references therein (4). In some research, age was proposed to be the principal hazard factor for the generation of tumor, and canine mammary tumors were frequently observed in dogs ranging from 8 to 11 years, whereas young canines were more susceptible to benign tumors (13, 14, 32). These results were again confirmed by this study. The prevalence of tumors has been increasing in the past 9–12 years, and most of them were malignant tumors. A higher prevalence of canine mammary tumor was noticed in pure breed dogs, and it was mostly represented by small-size dogs. Few literatures mentioned that male dogs suffered from mammary gland tumors, but this study showed that 2.58% (13/504) of CMTs occurred in the male dogs. It could be seen that 97.42% (491/504) happened to the female dogs, mainly non-spayed dogs, which might be closely related to the hormonal exposure of intact dogs. Hormonal exposure was identified as a hazard factor associated with CMT and steroid hormones, principally 17 beta-estradiol (E2), participated in cell proliferation by playing an antiapoptotic role in tumor progression (33, 34). Moreover, Schneider's study revealed that the incidence rates of CMTs in female dogs spayed before the first estrus were significantly lower than those in female dogs spayed after the first estrus (17). These findings were completely consistent with the results of the present investigation. The tumor size also passed for one of the major macro consequences interrelated to the deed of canine mammary tumor. According to the previous research, benign tumors are usually smaller than malignant tumors, and their prognosis may be better than that of large tumors (24, 32). In this study, tumor size ranged from 0.5 to 28 cm, with malignant tumors (4.33 ± 2.88 cm, mean ± SD) being bigger than benign tumors (3.06 ± 1.67 cm, mean ± SD), and the tumor size was proportional to the malignant grades, implying that other factors changed the tumor properties with an increase in tumor size. Furthermore, the incidence rate of tumor in the last two pairs of mammary glands was higher compared with other mammary glands, which was in agreement with the previous study (4). In addition, owing to the environmental differences, the morbidities of tumor in different regions could be variable. The prevalence of CMTs for the 5-year-total differed in different regions of China. The highest prevalence of occurrence was found in Northeast China, with the lowest in Southeast China, and it could be linked to regional development and the owner's animal breeding knowledge. In addition to environmental influences, our multiple linear regression analysis of canine mammary tumor histological qualities discovered that age of dog, tumor size, and tumor number all played a role in tumor properties.

## Conclusions

In summary, this retrospective study provided a unique opportunity to fully understand the main factors contributing to canine mammary tumor. In this study, the observation that a high number of malignant tumors are smaller than 1 cm suggests the need for a reconsideration of the size (T) parameter in the TNM system, and the result showed that canine mammary tumors occur more frequently in non-spayed dogs, suggesting that the early implementation of ovarian hysterectomy can reduce the incidence of canine mammary tumor in dogs. This study may also provide a methodology for the investigation and control of clinical risk factors for other tumors.

## Data Availability Statement

The original contributions presented in the study are included in the article/supplementary material, further inquiries can be directed to the corresponding author.

## Author Contributions

H-HZ devised, structured, and wrote the manuscript. All authors reviewed and corrected the manuscript.

## Funding

This work is supported by grants from the National Science Foundation of China (No. 31672616), the National Key R&D Program of China (No. 2016YFD0501000), the Natural Science Foundation of Jilin Province (No. 20210101002JC), and the Fundamental Research Funds for the Central Universities (No. JLUXKJC2021ZY09).

## Conflict of Interest

The authors declare that the research was conducted in the absence of any commercial or financial relationships that could be construed as a potential conflict of interest.

## Publisher's Note

All claims expressed in this article are solely those of the authors and do not necessarily represent those of their affiliated organizations, or those of the publisher, the editors and the reviewers. Any product that may be evaluated in this article, or claim that may be made by its manufacturer, is not guaranteed or endorsed by the publisher.
